# The engineered bladder patch with a three‐layer structure promotes the regeneration and functional recovery of the bladder in a rabbit model

**DOI:** 10.1002/btm2.70140

**Published:** 2026-04-21

**Authors:** Di Li, Pengchao Wang, Kaipeng Bi, Yang He, Jinxuan Zhang, Ziyan An, Zheng Wang, Linqing Ji, Jianye Li, Dawei Mu, Fei Liu, Weijun Fu, Shuwei Xiao

**Affiliations:** ^1^ Department of Urology Chinese PLA Air Force Medical Center, Air Force Medical University Beijing China; ^2^ Department of Urology Hainan Hospital of Chinese PLA General Hospital Sanya China; ^3^ Department of Urology Third Medical Center, PLA General Hospital Beijing China; ^4^ Medical School of PLA Beijing China; ^5^ School of Medicine, Nankai University Tianjin China; ^6^ Department of Urology Fifth Medical Center, PLA General Hospital Beijing China

**Keywords:** bladder tissue engineering, nerve regeneration, photo‐crosslinked hydrogel, silk fibroin scaffold, vascularization

## Abstract

The engineered bladder patch with a three‐layer structure was constructed from a bi‐layer silk fibroin scaffold (BSFS) and a methacrylated bladder acellular matrix hydrogel (BAMMAH) that carried induced vascular endothelial cells (VECs) and induced nerve cells (NCs). The patch was developed for bladder augmentation in a rabbit model. The prepared BSFS combined the rigidity of silk fibroin film with the elasticity of silk fibroin sponge, providing a support framework for the construction of engineered bladder. The synthesized BAMMAH exhibited a rapid gelling property, and 2% BAMMAH showed good rheological properties and an internal pore structure. Immunofluorescent staining and RT‐qPCR confirmed that the induction schemes for adipose‐derived mesenchymal stem cells (ADSCs) differentiation into VECs and NCs were feasible and stable. Immunofluorescence analysis demonstrated that incubation with omentum could promote the regeneration of the vascular network inside the engineered patch with a three‐layer structure (BSFS‐BAMMAH‐ADSCs‐VECs‐NCs). Animal experimental data showed that the engineered patch could promote the regeneration of the bladder wall and the recovery of bladder function. These results confirmed that the constructed engineered bladder patch with a three‐layer structure could regenerate the blood vessel network and neural reinnervation, providing a feasible method to solve the difficulties of vascular network and neural innervation in bladder tissue engineering research.

## Graphical abstract

A brief schematic drawing of the design and application of the engineered bladder patch with a three‐layer structure.

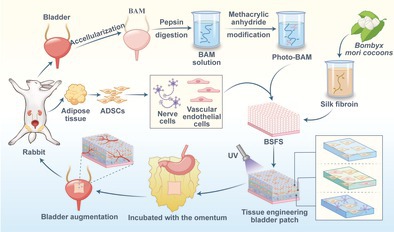

**Translational Impact Statement**

In this study, the engineered bladder patch with a three‐layer structure constructed by layer‐by‐layer assembly technology regenerated internal blood vessel and neural networks, achieving the regeneration of bladder wall structure and the restoration of bladder physiological function, providing a feasible method for constructing the engineered bladder with a blood vessel network and neural innervation. The findings are significant for speeding medical translation and clinical application of the engineered bladder.

## 1 INTRODUCTION

The bladder is an important organ whose main function is to store urine at a low and stable pressure and empty urine in a controlled manner.^1^ However, congenital and acquired defects such as myelomeningocele, bladder exstrophy, trauma, malignant tumors, and so on may cause bladder dysfunctions and even damage kidney function.^2^ Bladder reconstruction and urinary diversion are necessary to restore bladder function and protect kidney function. The gastrointestinal tract is often used for bladder reconstruction and urinary diversion, which is still the gold standard in clinical therapy.^3^ Nevertheless, enterocystoplasty is commonly associated with a series of complications, such as metabolic disturbance, mucus production, anastomotic malignancy, stone formation, fibrosis, and long‐term bacteriuria.^4^, ^5^ New methods and technologies are urgently needed to reconstruct bladder defects and restore bladder function in clinical practice.

With the rapid development and innovation of tissue engineering technology, tissue‐engineering bladder, based on scaffold materials and seed cells, is expected to provide new treatment techniques and solutions for the repair and reconstruction of bladder defects.^6^ The scaffold materials used in bladder tissue engineering mainly include synthetic and natural materials.^7^ Among them, natural biomaterials are attractive scaffold materials due to their excellent biodegradability and biocompatibility, as well as their ability to mimic the characteristics of the extracellular matrix (ECM).^8^ In our previous research, it had been confirmed that scaffold materials prepared using silk fibroin (SF) had excellent elasticity, toughness, and good biocompatibility, which could be used as scaffold materials for bladder tissue engineering.^9^

Moreover, the decellularized matrix materials in natural biomaterials were widely used in tissue engineering research because they had removed cellular components that could cause immune responses, while retaining the ECM structure and components of organs or tissues themselves.^10^ Currently, the photo‐cross‐linked decellularized matrix hydrogels have been considered one of the most suitable choices for tissue engineering scaffold materials because of the spatiotemporal controllability of the photo‐cross‐linking method and tissue‐specific ECM components.^11^, ^12^, ^13^ There were relatively few reports on the photo‐cross‐linked bladder acellular matrix (Photo‐BAM). Based on previous research on self‐assembled BAM hydrogel, we prepared a photo‐BAM hydrogel for bladder tissue engineering research and verified its feasibility and safety.

Adipose‐derived mesenchymal stem cells (ADSCs), as seed cells, have attracted much attention in bladder tissue engineering research due to their wide tissue sources, convenient sampling, and multi‐directional differentiation potential.^14^, ^15^ ADSCs could promote the regeneration of blood vessels and nerves through paracrine secretion and multipotent differentiation ability. Previous studies had reported that ADSCs could promote neovascularization by paracrine factors and directly differentiate into endothelial cells, and also could promote nerve regeneration by directly differentiating into nerve cells and secreting growth factors.^16^, ^17^ Similarly, it was crucial for tissue engineering grafts to rapidly regenerate blood vessels and nerves after transplantation into the body in bladder tissue engineering research. Regenerated blood vessels could deliver oxygen and nutrients to the grafts, thereby preventing fiber contraction, perforation, and urine leakage.^18^ Moreover, the regeneration of nerve cells was also essential for the recovery of bladder function.^19^ Our previous research had confirmed that tissue‐engineered bladder scaffolds constructed using only ADSCs as seed cells could promote vascular regeneration. However, they could not fully restore bladder function, which might be due to the insufficient nerve regeneration.^9^, ^20^

In this study, the isolated and cultured ADSCs were induced to differentiate into vascular endothelial cells and nerve cells, serving as seed cells. Meanwhile, the prepared photo‐BAM hydrogel (methacrylated bladder acellular matrix hydrogel, BAMMAH) was used as the carrier to transport different types of seed cells. Additionally, the bi‐layer silk fibroin scaffold (BSFS) was prepared using SF solution as the supporting skeleton. Using the rapid photopolymerization and curing characteristics of the photo‐BAM hydrogel, and in combination with the distinct structural features of each layer of the bladder wall, the engineered bladder patch was constructed in three layers through the layer‐by‐layer assembly technique on the BSFS. The constructed patch was incubated with the omentum to achieve the vascularization, and then bladder augmentation was performed to evaluate the effect of the engineered bladder patch in vivo.

## 2 RESULTS

### 2.1 Preparation and characterization of BSFS and BAMMAH

**FIGURE 1:**
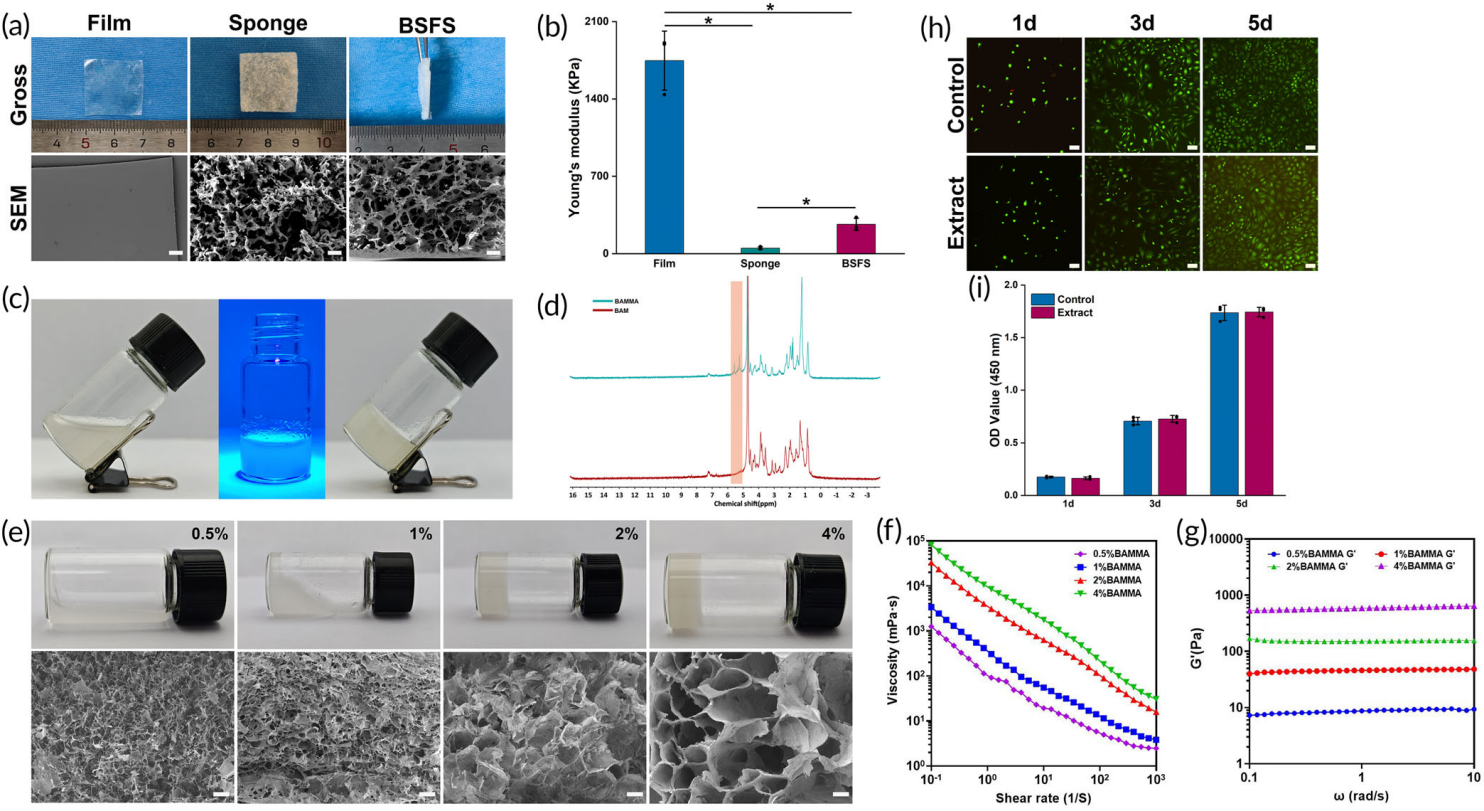
Characterizations of the BSFS and BAMMAH and cytotoxicity test of the BSFS‐BAMMAH. (a) Gross photos and SEM micrographs of the BSFS; Scale bar = 200 μm. (b) Tensile test (*n* = 3). (c) The preparation of BAMMAH. (d) ^1^H NMR spectroscopy. (e) Gross and SEM photographs of the BAMMAHs; Scale bar = 100 μm (f) Shear rate/viscosity of the rheological analysis. (g) Frequency/storage modulus of the rheological analysis. (h) Live/dead staining (Green: live cells. Red: dead cells); Scale bar = 100 μm. (i) CCK‐8 test (*n* = 3). (*p*‐values were calculated using a one‐way analysis of variance with Bonferroni post hoc test, *p < 0.05).

The gross observation showed that the prepared SF film was thin and tough, while the SF sponge was soft and elastic. Moreover, the scanning electron microscopy (SEM) images confirmed that the surface structure of SF film was dense without any pore structures, while the surface structure of SF sponge was loose with a large number of pore structures. The prepared BSFS not only had the toughness of SF film but also the elasticity of SF sponge (Figure 1a). In the tensile test, the Young's modulus of SF film (1747.13 ± 267.53 KPa) was significantly higher than that of SF sponge (50.00 ± 11.69 KPa) and BSFS (265.74 ± 54.66 KPa) (*p* < 0.05), and the Young's modulus of BSFS was significantly higher than that of SF sponge (*p* < 0.05) (Figure 1b). H&E, Masson, and DAPI staining showed that the prepared BAM removed the nucleus and retained the collagen structure (Figure S1A). In addition, the DNA content testing confirmed that the residual DNA in the BAM (80.67 ± 6.46 ng/mg) was significantly less than that in the native bladder tissue (688.93 ± 27.21 ng/mg, *p* < 0.05) (Figure S1B). The prepared BAMMA solution with 0.25% (vol/vol) LAP was exposed to 405 nm UV light for 1 min to obtain the BAMMAH (Figure 1c). Moreover, ^1^H NMR spectroscopy showed that two new peaks (5.25 and 5.53 ppm) appeared in the BAMMA (Figure 1d). The gross photographs demonstrated that the low concentration BAMMAHs (0.5% and 1%) were semisolid and the high concentration BAMMAHs (2% and 4%) presented a solid form. Additionally, the SEM results confirmed that the structures of the low concentration BAMMAHs (0.5% and 1%) were loose and collapsed without obvious pore structures, while a large number of pore structures were visible in the high concentration BAMMAHs (2% and 4%) (Figure 1e). Rheological results showed that BAMMAHs with different concentrations exhibited a good shear dilution characteristic, and the viscosity gradually decreased with an increase in shear rate (Figure 1f). At the same time, when the concentrations of BAMMAH were increased, the storage modulus was also increased (Figure 1g). Live/dead staining indicated that the ADSCs cultured in the extract of BSFS‐BAMMAH had good cell viability at 1, 3, and 5 days (Figure 1h). Furthermore, CCK‐8 testing confirmed that there was no significant difference in absorbance at 450 nm between the control group and the BSFS‐BAMMAH extract group at different time points (Figure 1i).

### 2.2 Induced differentiation of ADSCs

**FIGURE 2:**
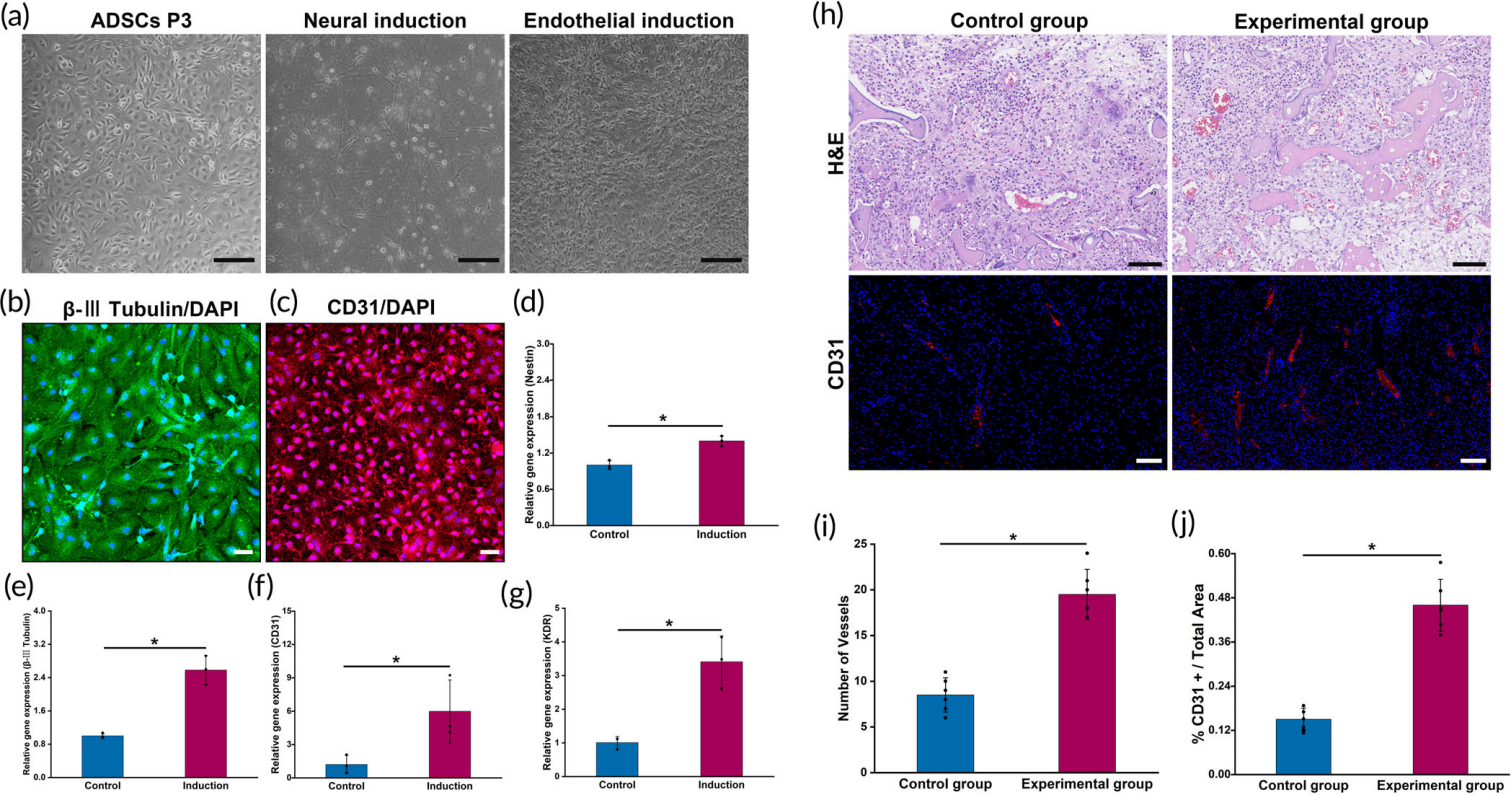
Induced differentiation of ADSCs and vascularization evaluation of the constructed engineered bladder patches embedded with omentum. (a) The cell morphology of ADSCs, induced‐NCs, and induced‐VECs. Scale bar = 500 μm. (b) Immunofluorescence staining of induced‐NCs (Green: β‐III Tubulin, blue: DAPI); Scale bar = 50 μm. (c) Immunofluorescence staining of induced‐VECs (Red: CD31, blue: DAPI); Scale bar = 50 μm. (d) Nestin gene (*n* = 3) (*p < 0.05). (e) β‐III Tubulin gene (*n* = 3). (f) CD31 gene (*n* = 3). (g) KDR gene (*n* = 3). (h) H&E and immunofluorescence staining of the constructed engineered bladder patches embedded with omentum (Red: CD31, blue: DAPI); Scale bar = 100 μm. (i) The number of CD31‐positive vessels (*n* = 6). (j) The positive area of CD31 (*n* = 6). (*p*‐values were calculated using a two‐tailed Student's *t*‐test, *p < 0.05).

The flow cytometry results confirmed that the isolated cells had positive expression for CD29 and CD44, and negative expression for CD34 and CD45 (Figure S2). The isolated third‐passage ADSCs showed a spindle‐shaped morphology. After 10 days of neural induction differentiation, some cell bodies showed long and thin structures resembling axons. After endothelial induction differentiation for 10 days, the cell morphology appeared as flattened oval or spindle‐shaped, and adjacent cells were tightly connected (Figure 2a). Immunofluorescent staining indicated that the positive expression of β‐III Tubulin could be observed in the cells of neural induction differentiation, and the CD31 positive expression could be found in the cells of endothelial induction differentiation (Figure 2b,c). At 10 days of neural induction differentiation, the relative expression level of Nestin and β‐III Tubulin in the induction group was significantly higher than that in the control group (*p* < 0.05) (Figure 2d,e). At 10 days of endothelial induction differentiation, the relative expression level of CD31 and KDR in the control group was significantly lower than that in the induction group (*p* < 0.05) (Figure 2f,g).

### 2.3 The distribution of the cells in the engineered bladder patch and vascularization evaluation of the constructed engineered bladder patches embedded with omentum

In the engineered bladder patch of the experimental group, H&E staining revealed the presence of BSFS, BAMMAH, and seed cells (Figure S3A). The SEM results and DAPI staining confirmed that the seed cells were distributed on both the surface and inside of the patch (Figure S3B,C). H&E and immunofluorescent staining indicated that the vascular structures could be observed in the engineered bladder patches embedded with omentum (Figure 2h). Furthermore, the number of CD31‐positive vessels (19.50 ± 2.74 vs. 8.50 ± 1.87) and the positive area of CD31 (0.46 ± 0.07% vs. 0.15 ± 0.03%) in the experimental group was significantly higher than that in the control group (*p* < 0.05) (Figure 2i,j).

### 2.4 Imaging examination and stone analysis

**FIGURE 3:**
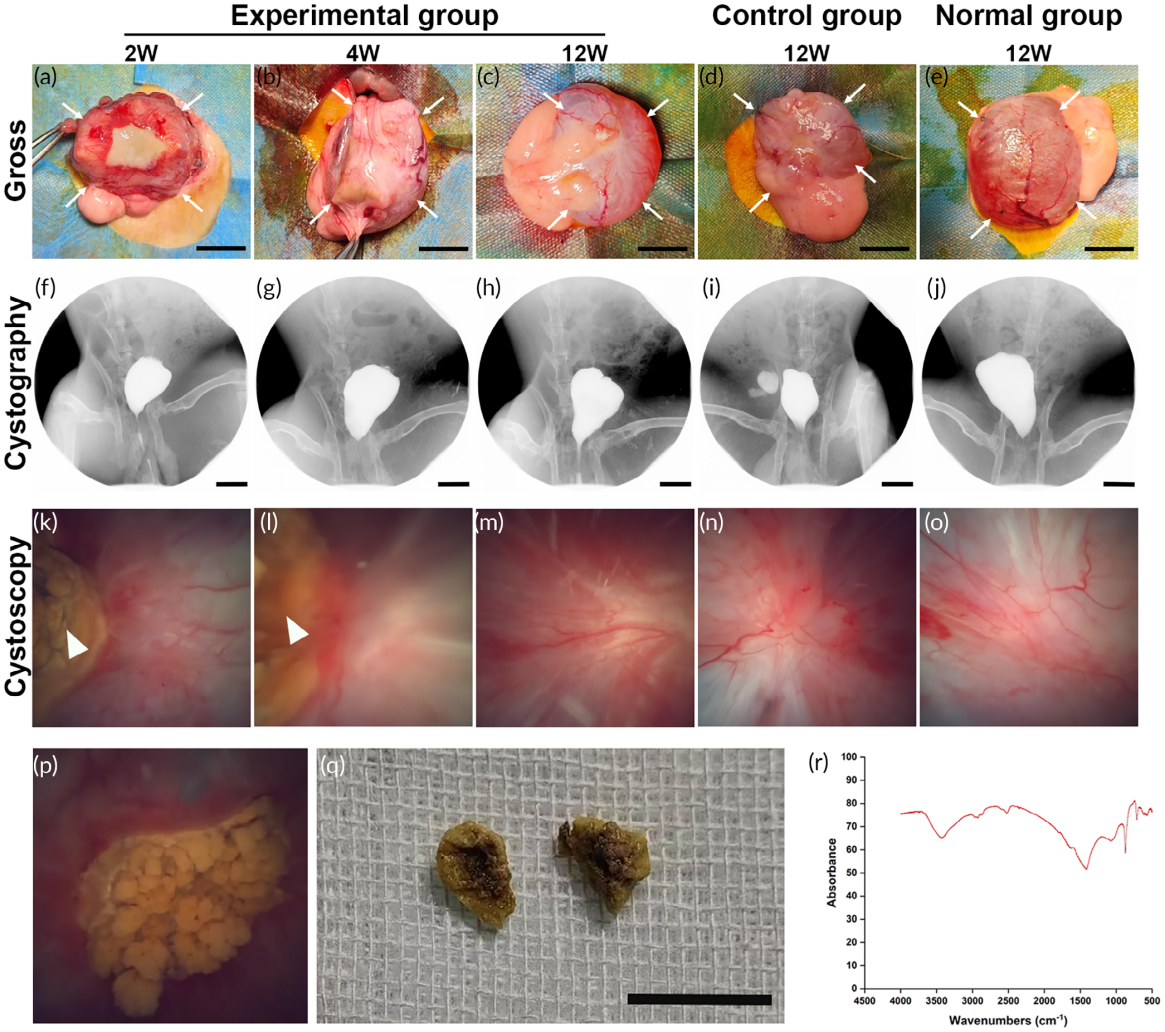
Imaging examination and stone analysis. (a–e) Gross photographs of the patches in the three groups (White arrows indicate the bladder reconstruction area); Scale bar = 1 cm. (f, j) Cystography; Scale bar = 1 cm. (k–o) Cystoscopy (White triangles mark the stones). (p) Stone morphology in cystoscopy. (q) The core of the stone; Scale bar = 1 cm. (r) Stone spectrum.

At 2 weeks after surgery, the overall shape of the transplanted engineered bladder patch was visible and clearly demarcated from the surrounding normal bladder tissue, and the BSFS was not degraded in the experimental group (Figure 3a). At 4 weeks, the BSFS was partially degraded, and the boundary between the transplanted patch and the surrounding bladder tissue was still visible in the experimental group (Figure 3b). At 12 weeks, the BSFS in the experimental and control groups was completely degraded, and the boundary between the transplanted patches and the surrounding bladder tissue was blurred and integrated in all three groups (Figure 3c–e). In the experimental group, cystography confirmed that the dome structure of the bladder had disappeared at 2 weeks (Figure 3f) and the apex of the bladder was flat at 4 weeks (Figure 3g). At 12 weeks, the bladder morphology in both the experimental and normal groups was normal, with a visible bulging bladder crest structure (Figure 3h,j). However, in the control group, the bladder top wall was relatively flat (Figure 3i). In addition, cystography revealed that leakage of the contrast agent was not observed at any time point in each group. Cystoscopy confirmed the presence of bladder stones at 2 and 4 weeks in the experimental groups (Figure 3k,l), and the stones were located at the core of the transplanted patches (Figure 3p). In addition, at 2 weeks, proliferative follicles were visible around the stone, and the transplanted patch had a clear boundary with the surrounding normal bladder mucosa (Figure 3k). At 4 weeks, the proliferative follicles around the stone had disappeared, and the surrounding mucosa became pale with a clear boundary from normal bladder mucosa (Figure 3l). At 12 weeks, stones were not found in the center of the transplanted patches in all three groups, and the boundary between the regenerated bladder mucosa and the surrounding normal bladder mucosa was unclear (Figure 3m–o). When the bladder stones were removed and opened, degraded BSFS and sutures could be observed in the core of the stones (Figure 3q). Furthermore, the analysis of stone composition confirmed that the main component of bladder stones was calcium carbonate (Figure 3r).

### 2.5 Histological examination

**FIGURE 4:**
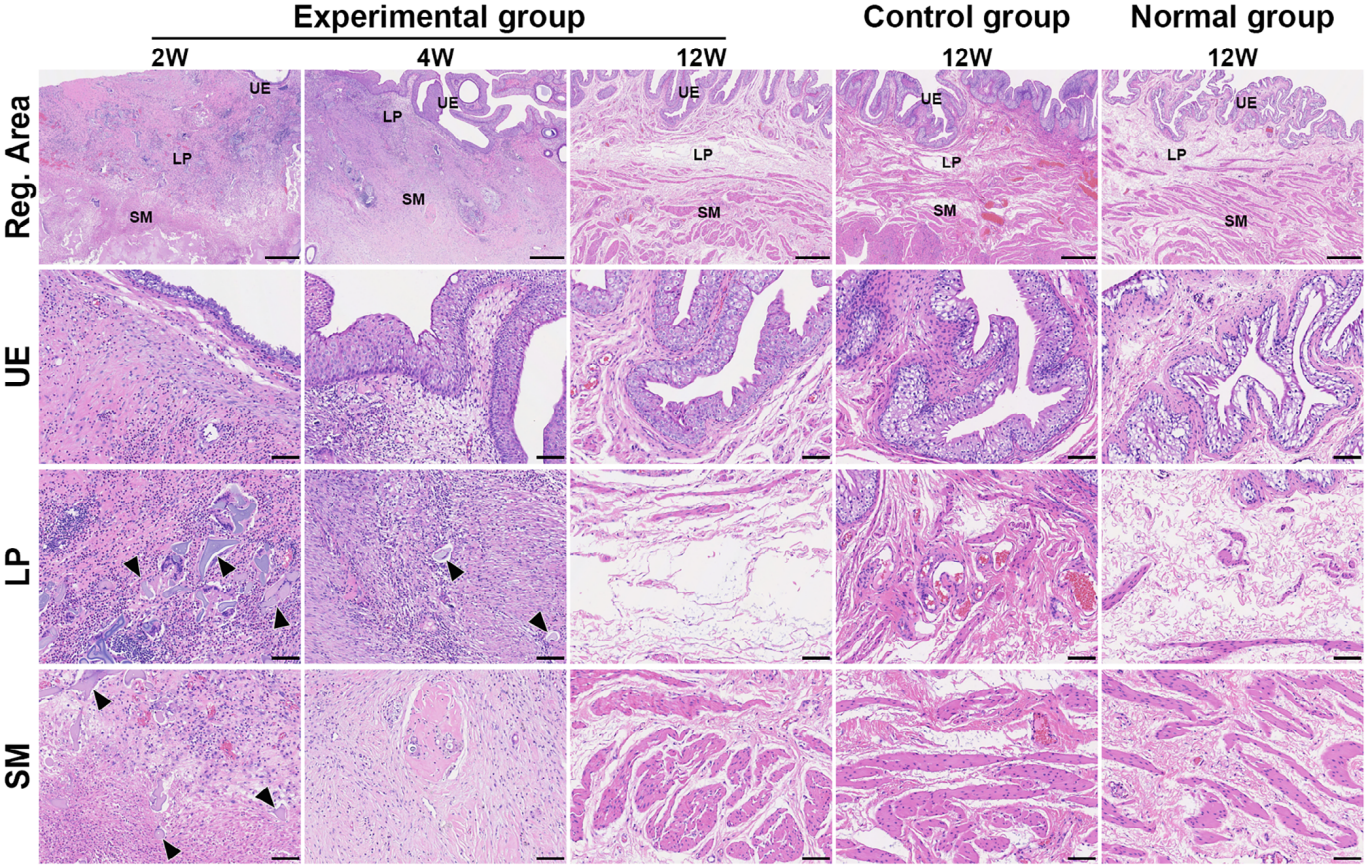
Histological examination of the regenerated bladder wall in the three groups. LP, lamina propria; SM, smooth muscle; UE, urothelium. Black triangles indicate the residual BSFS. Reg. Area: Scale bar = 600 μm; UE, LP, SM: Scale bar = 100 μm.

H&E staining revealed that the multi‐layered structure of the bladder wall was observable in all three groups, including the mucosal layer, the lamina propria, and the smooth muscle layer, at 12 weeks post‐surgery. At the same time, multiple layers of urothelial cells could be observed in the mucosal layer, vascular structures could be detected in the lamina propria, and bundles of smooth muscle tissue could be seen in the smooth muscle layer. However, the multi‐layered structure of the bladder wall was not obvious at 2 and 4 weeks after surgery in the experimental group. In addition, the residual BSFS could also be observed. At 2 weeks, the urothelial cells in the mucosal layer were not continuous, and no obvious vascular structure was observed in the lamina propria, and only a small amount of smooth muscle tissue was present in the smooth muscle layer. At 4 weeks, proliferative urothelial cells could be observed in the mucosal layer, a few vascular structures could be shown in the lamina propria, and a small amount of bundled smooth muscle tissue could be detected in the smooth muscle layer (Figure 4).

### 2.6 Immunofluorescence analyses

**FIGURE 5:**
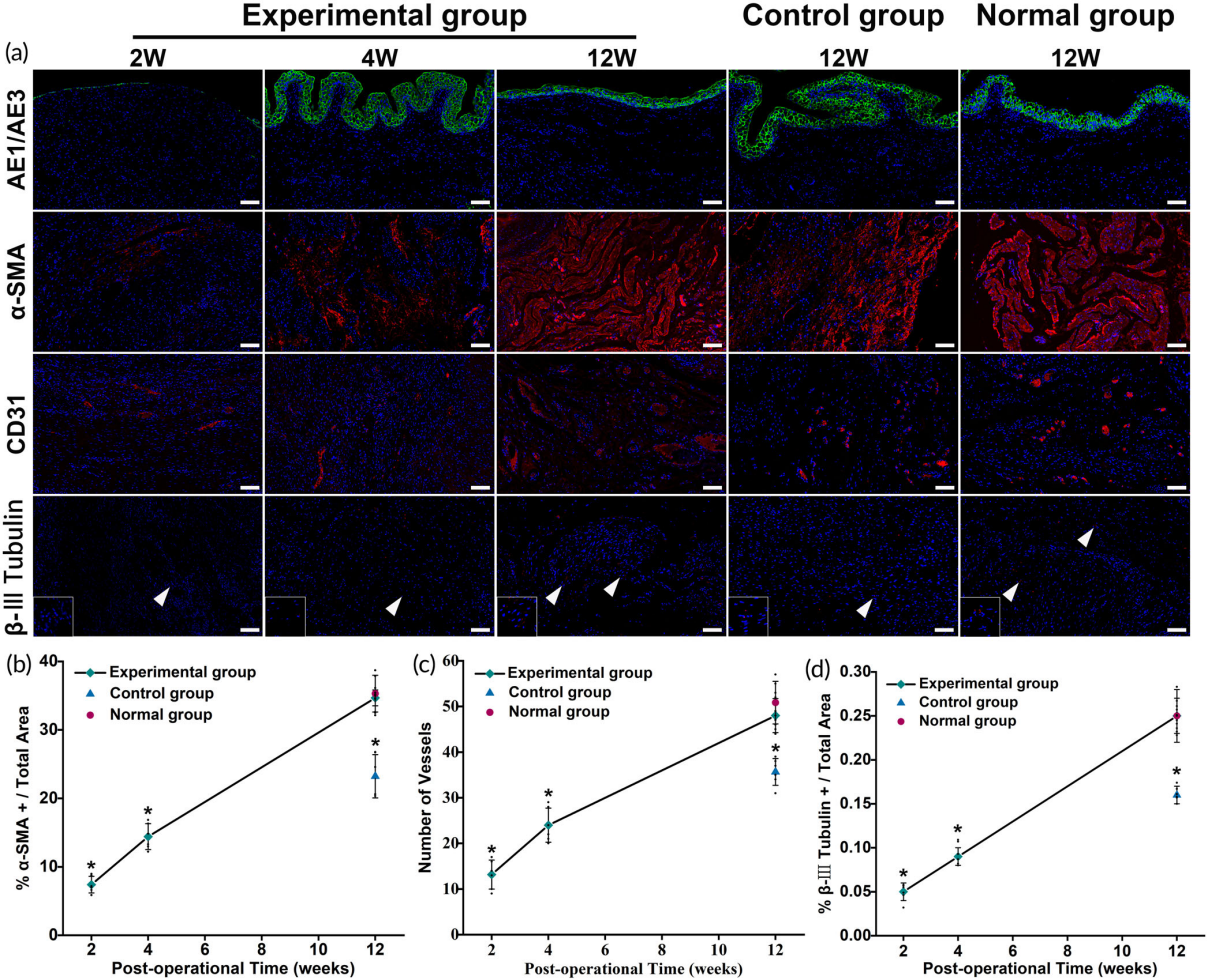
Immunofluorescence analyses of the regenerated bladder tissue in the three groups. (a) AE1/AE3: Green; α‐SMA: Red, CD31: Red, β‐III Tubulin: Red, DAPI: Blue; White triangles mark the nerve cells; Scale bar = 100 μm. (b) The positive area of α‐SMA (*n* = 6). (c) The number of CD31‐positive vessels (*n* = 6). (d) The positive area of β‐III Tubulin (*n* = 6). (*p*‐values were calculated using a one‐way analysis of variance with Bonferroni post hoc test, *p < 0.05).

Immunofluorescence staining could observe the expression of AE1/AE3, α‐SMA, CD31, and β‐III Tubulin in different groups at different points (Figure 5a). In the experimental group, the positive area of α‐SMA was significantly larger at 12 weeks (34.67 ± 1.15%) after surgery than that at 2 (7.40 ± 1.22%) and 4 (14.41 ± 1.89%) weeks (*p* < 0.05). At 12 weeks after surgery, the positive area of α‐SMA in the experimental group was higher than that in the control group (23.23 ± 3.16%, *p* < 0.05), which was no different from that in the normal group (35.30 ± 2.69%) (Figure 5b). The number of CD31‐positive vessels at 2 (13.17 ± 3.19) and 4 (24.00 ± 3.74) weeks was significantly less than that at 12 weeks (48.00 ± 3.74) in the experimental group (*p* < 0.05). The number of CD31‐positive vessels in the experimental group showed no significant difference from the normal group (50.83 ± 4.67), which was significantly higher than that in the control group (35.67 ± 2.94, *p* < 0.05) at 12 weeks (Figure 5c). At 12 weeks, the positive area of β‐III Tubulin in the experimental group (0.25 ± 0.02%) was significantly larger than that in the control group (0.16 ± 0.01%, *p* < 0.05), and there was no difference compared to the normal group (0.25 ± 0.03%). The positive area of β‐III Tubulin at 2 (0.05 ± 0.01%) and 4 (0.09 ± 0.01%) weeks was significantly less than that at 12 weeks (*p* < 0.05) in the experimental group (Figure 5d).

### 2.7 Urodynamic evaluations

**FIGURE 6:**
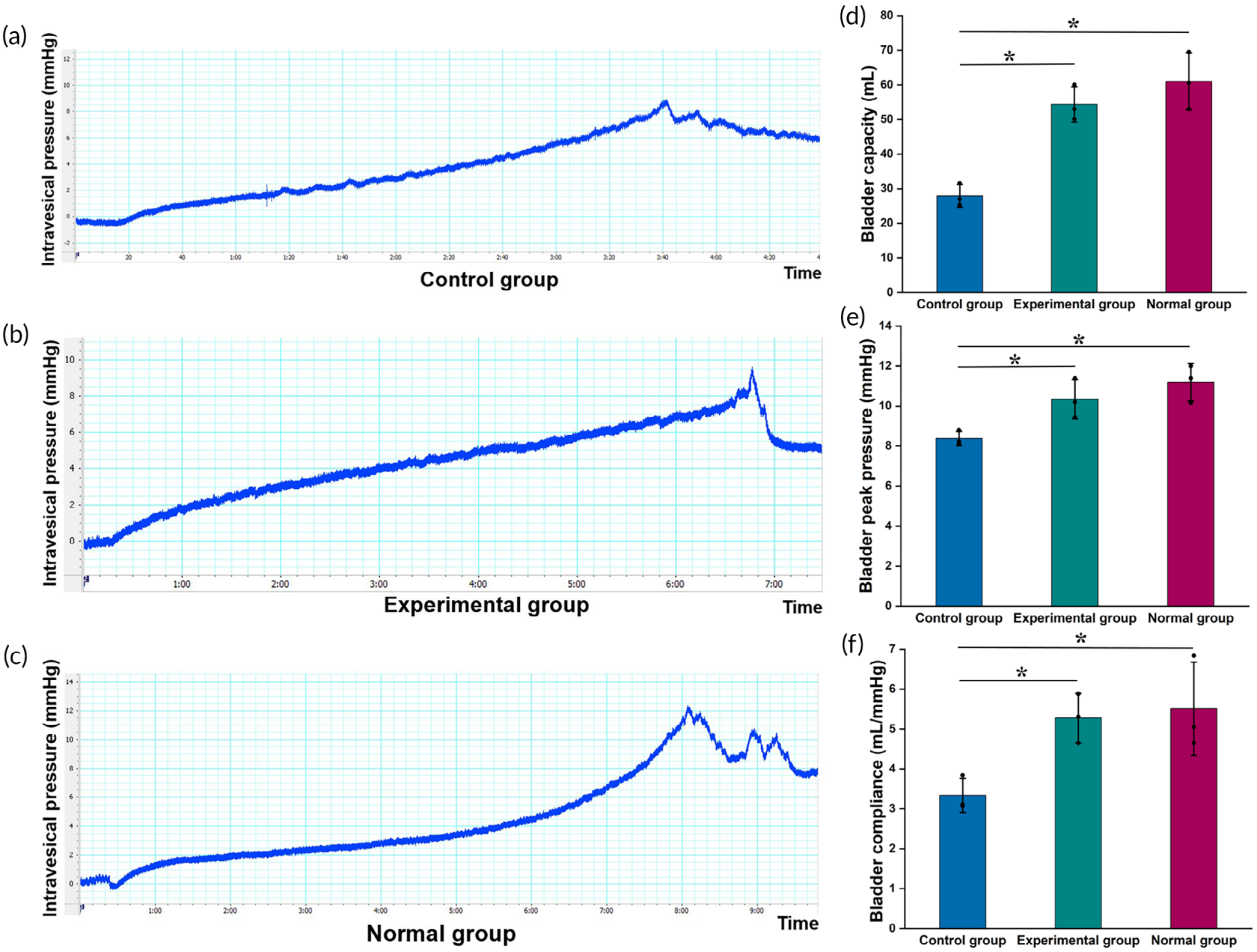
Urodynamic evaluations. (a) Urodynamic curve of the control group. (b) Urodynamic curve of the experimental group. (c) Urodynamic curve of the normal group. (d) Bladder capacity in the three groups (*n* = 3). (e) Bladder peak pressure in the three groups (*n* = 3). (f) Bladder compliance in the three groups (*n* = 3). (*p*‐values were calculated using a one‐way analysis of variance with Bonferroni post hoc test, *p < 0.05).

At 12 weeks after surgery, the urodynamics were recorded to examine the physiological function of the bladder (Figure 6a–c). The bladder capacity and peak pressure between the experimental group (54.37 ± 5.09 mL, 10.35 ± 0.99 mmHg) and normal group (61.03 ± 8.26 mL, 11.19 ± 0.94 mmHg) were of no significant difference, while the bladder capacity and peak pressure in the control group (27.97 ± 3.26 mL, 8.38 ± 0.36 mmHg) were lower than that in the experimental and control groups (*p* < 0.05) (Figure 6d,e). The bladder compliance in the experimental group (5.28 ± 0.62 mL/mmHg) was higher than that in the control group (3.34 ± 0.43 mL/mmHg) (*p* < 0.05), and there was no difference compared to the normal group (5.51 ± 1.17 mL/mmHg) (Figure 6f).

## 3 DISCUSSION

Research on bladder tissue engineering primarily involves the selection of scaffold materials, the proliferation, and differentiation of seed cells, ensuring a sufficient blood supply, and neural regulation.^21^, ^22^, ^23^ Among them, the engineered bladder constructed with scaffold materials and seed cells simulates the bladder wall structure. A sufficient blood supply is essential for the survival of the engineered bladder transplanted into the body. Neural regulation is the key to the functional performance of the engineered bladder.

The selection of scaffold materials is particularly important in the construction of an engineered bladder. Ideal scaffold materials must have good mechanical properties to provide support, good biocompatibility to avoid rejection reactions, and simulate the microenvironment of the ECM to facilitate the adhesion, growth, and proliferation of seed cells.^24^, ^25^ Compared to the previous study,^9^ in this study, we prepared a larger volume BSFS, tested its material properties, and confirmed the stability of the BSFS preparation system. The BSFS, composed of SF film and SF sponge, combined the rigidity of SF film with the elasticity of SF sponge, providing a support framework for the construction of an engineered bladder. At the same time, the prepared BSFS not only possessed the toughness of SF film but also had the pore structure of SF sponge, which was conducive to surgical suturing and the implantation of seed cells. Hydrogels, three‐dimensional networks of cross‐linked hydrophilic polymers characterized by high water content and excellent biocompatibility, mimic the natural ECM.^26^ ^1^H NMR spectroscopy confirmed that the prepared BAMMMA successfully cross‐linked with methacrylate vinyl and exhibited photo‐cross‐linking property. Compared with the self‐cross‐linked BAM hydrogel,^20^ the prepared photo‐BAM hydrogel exhibited a rapid gelling property, which could be quickly gelled within 1 min and could be configured with different concentrations of BAMMAHs as required. Meanwhile, the different concentrations of BAMMAHs had different rheological properties and internal pore structures. Based on the gross photos, SEM, and rheological results of BAMMAHs with different concentrations (0.5%, 1%, 2%, 4%), the 2% BAMMAH was used as a seed cell carrier to rapidly construct a multi‐layer engineered bladder.

Seed cells are the foundation of bladder tissue engineering research. ADSCs were widely used in bladder tissue engineering research due to their easy accessibility, high quantity, and more autocrine and multi‐directional differentiation potential.^27^ Our previous studies had confirmed that ADSCs could promote the regeneration of smooth muscle, blood vessels, and nerves.^9^, ^20^ In this study, based on the previous literature research,^28^, ^29^ our team explored neural induction and vascular endothelial induction schemes suitable for ADSCs. The gross photos showed that the morphology of axons and endothelial cells could be observed, respectively, after neural induction differentiation and vascular endothelial induction differentiation in the ADSCs. Additionally, immunofluorescence staining and RT‐qPCR could detect the expression of proteins and genes related to nerve cells (NCs) and vascular endothelial cells (VECs) in the induced differentiation of ADSCs. These results confirmed that the neural induction and vascular endothelial induction schemes were feasible and reliable. NCs and VECs induced by ADSCs were used as seed cells to construct a multi‐layered engineered bladder containing cells with different functions.

The normal structural anatomy of the bladder wall consists of four layers: mucosal layer (urothelium), submucosal layer (lamina propria), smooth muscle layer, and serosal layer.^30^ The mucosal layer is a unique structure of the urinary system that can protect the lamina propria from the erosion of the urine environment. The lamina propria is mainly composed of fibrous and bundled collagen, which contains rich vascular structures and provides nutritional supply to the mucosal layer. The smooth muscle layer is the core structure of the bladder wall. The circular and longitudinal smooth muscle bundles in the smooth muscle layer coordinate contraction and relaxation through nerve conduction, thereby achieving bladder filling and urination function.^31^ In this study, layer‐by‐layer assembly technology was used to construct an engineered bladder wall to simulate the structural characteristics of each layer of the bladder wall. The prepared BSFS was used as the support framework, and the smooth muscle layer, lamina propria, and mucosal layer were assembled in sequence from bottom to top. The rapid cross‐linking of BAMMA carrying ADSCs and induced differentiated neural cells served as the bottom layer, the BAMMA carrying ADSCs and induced differentiated vascular endothelial cells served as the middle layer, and the BAMMA carrying ADSCs alone served as the upper layer. H&E staining, SEM, and DAPI staining showed the distribution of cells on both the surface and inside of the constructed engineered bladder patch.

The formation of blood vessels is a critical goal in tissue engineering for regenerative medicine.^32^ Meanwhile, vascularization remains a major challenge in tissue engineering, especially for large and thick tissues such as bladder tissue.^33^ In order to construct the vascular network inside the engineered bladder, various vascularization methods were used in this study. Previous research had confirmed that the prepared BAM effectively preserved various growth factors, such as vascular endothelial growth factor, platelet‐derived growth factor BB, keratinocyte growth factor, and so on.^34^ When the BAM was digested, dissolved, and polymerized to form a hydrogel, growth factors were still retained in the BAM hydrogel.^35^ In this study, the prepared BAMMAH was used as the scaffold material to transport seed cells, which could not only provide an ECM microenvironment to promote the proliferation and differentiation of seed cells, but also could promote angiogenesis by the growth factors that may remain in the BAMMAH. Moreover, the induced‐VECs and ADSCs were used as seed cells to participate in the construction of the middle layer of the engineered bladder patch. As is well known, VECs are the primary cell type that forms the inner lining of blood vessels, serving as a critical interface between the bloodstream and surrounding tissues. VECs contribute to the establishment of initial vascular structures and facilitate functional blood flow.^36^ At the same time, the ADSCs could promote neovascularization by paracrine factors and directly differentiate into endothelial cells.^16^ The induced‐VECs and ADSCs in the middle layer of the engineered bladder patch played a key role in promoting the formation of its internal vascular network. Additionally, omentum incubation may promote the formation of new blood vessels and tissue remodeling within the tissue‐engineered bladder. Numerous research results have confirmed this point.^9^, ^20^, ^37^ The research results confirmed that the number of newly formed blood vessels inside the engineered bladder patch in the experimental group was significantly higher than that in the control group after 1 week of incubation with omentum. At 12 weeks after bladder augmentation, the number of blood vessels in the experimental group was also significantly higher than that in the control group. In summary, the synergistic effect of scaffold materials, seed cells, and omentum incubation in this study enabled the construction of a vascular network within the engineered bladder patch.

The purpose of bladder tissue engineering repair and reconstruction research is to restore the complete morphology of bladder tissue, regenerate the bladder wall structure, and recover the physiological function of the bladder.^21^, ^38^ In this study, the gross photos and cystography showed that the bladder morphology of the experimental group and normal group was fully recovered at 12 weeks after surgery, while the bladder top wall was relatively flat in the control group. The possible reason for the incomplete recovery of bladder morphology in the control group was due to insufficient regeneration of the smooth muscle layer. Furthermore, H&E staining revealed that regeneration of bladder wall structures, including the urothelium, lamina propria, and smooth muscle layer, was observed in all three groups. Among them, H&E and immunofluorescence staining could detect multi‐layer continuous urothelial cells in all three groups. Based on previous literature reports,^39^, ^40^ we hypothesized that the regenerated urothelium of engineered bladder patches in all three groups was the result of very rapid natural urothelial regeneration, and the ADSCs may be involved in regulating the remodeling process of the uroepithelium. This was consistent with our previous researches.^9^, ^20^ Moreover, immunofluorescence staining could observe a large number of blood vessels in the lamina propria of the engineered bladder patches in the experimental and control groups, and the fluorescence quantitative analysis results identified that the number of blood vessels in the experimental and normal groups was significantly higher than that in the control group. Induced‐VECs directly participated in the formation of blood vessels, leading to this difference. In addition, immunofluorescence staining and quantitative analysis confirmed that the regenerated smooth muscle cells in the control group were significantly fewer than those in the experimental group and the normal group. The induced differentiation of ADSCs promoted the regeneration of smooth muscle cells, and the increased blood supply in both the experimental and normal groups facilitated the proliferation and induced differentiation of ADSCs, contributing to this difference. However, the bladder stones could be observed in the control and experimental groups at 2 and 4 weeks, while there were no bladder stones in the normal group. Previous studies reported that bladder stones were a common phenomenon in bladder augmentation with biodegradable scaffolds, in which the accumulation of degraded material could serve as a nidus for calculus formation.^9^, ^41^, ^42^ In this study, with the formation of the internal bladder wall structure of the engineered bladder patches in the control and experimental groups, the BSFS framework was gradually degraded. At the same time, with the degradation of BSFS and surgical sutures, as well as the deposition of urine within the bladder, cystoscopy revealed the formation of stones at the center of the engineered bladder patches at 2 and 4 weeks. Nevertheless, at 12 weeks after surgery, no obvious stones were observed in all three groups in cystoscopy. We considered that the stones might have fallen off the surface of the patches during the regeneration and remodeling of the internal structure of the engineered bladder patches. In addition, the recovery of bladder physiological function relies on the regeneration of the bladder wall, and neural reinnervation is the key. However, one of the greatest hurdles in bladder transplantation is the need for full neural reinnervation. Even if vascular anastomosis is achieved, inadequate nerve regeneration may lead to detrusor underactivity, urinary retention, or incontinence.^43^ Our previous research results also confirmed that the constructed engineered bladder patches did not fully restore the physiological function of the bladder due to insufficient nerve regeneration.^9^, ^20^ In this study, a large number of nerve cells were observed in the smooth muscle layer of the engineered bladder patch in the experimental group, with no significant difference compared to the normal group. Moreover, the urodynamic results showed no significant difference in bladder capacity, bladder peak pressure, and bladder compliance between the experimental and normal groups. Therefore, the regenerated nerve cells inside the engineered bladder patch in the experimental group promoted the recovery of bladder physiological function by coordinating the contraction and relaxation of smooth muscle cells. Whereas, all the experimental animals used in this study were male, which represented the limitation of our findings on female physiology. In this male model, the engineered bladder patch with a three‐layer structure exhibited excellent abilities for bladder regeneration and functional recovery, providing a feasible method for constructing an engineered bladder with a blood vessel network and neural innervation. Future studies are essential and planned to investigate its efficacy in female animal models, which will be a crucial step toward the medical translation and clinical application of the engineered bladder.

However, due to the interference of BSFS and BAMMAH, fluorescence staining was unable to display the induced‐VECs and induced‐NCs clearly, making it difficult to fully visualize the three‐layer structure of the engineered bladder patch by fluorescence staining. Meanwhile, at 2 weeks after bladder augmentation, the bladder stones could be observed in the core of the engineered bladder patches in the experimental and control groups. How to further optimize the selection and structure of the scaffold materials was also a problem that we need to solve in our future research. In addition, although a large number of nerve cells could be detected inside the engineered bladder patch of the experimental group, the mechanism by which the regenerated nerve cells connected and transmitted signals to the bladder's own neural network had not been fully elucidated. Despite the engineered bladder patch with a three‐layer structure having achieved bladder wall regeneration and functional recovery, the complexity of clinical translation cannot be ignored. Large‐scale, standardized production remains a technical bottleneck to achieving clinical accessibility. Therefore, there is still a long way to go to achieve clinical translation.

## 4 MATERIALS AND METHODS

### 4.1 Fabrication of the BSFS

The BSFS was constructed from silk fibroin film and silk fibroin sponge according to our previous study.^9^ Briefly, the *Bombyx mori cocoons* were cut into dime‐sized pieces and boiled in 0.02 M sodium carbonate to remove the sericin. The SF was dissolved in 9.3 M lithium bromide (Sigma) and then dialyzed and centrifuged to obtain the SF aqueous solution (8% wt/vol). Add 2 mL of 8% wt/vol SF aqueous solution into each well of the 6‐well plate, and dry overnight in the oven at 65°C to obtain the SF films. The structure of BSFS was obtained by preparing the SF sponge on the surface of the SF film. The 3 mL of 8% wt/vol SF aqueous solution were evenly dripped onto the surface of the SF film. Then 6 g sieved granular NaCl (300 μm, average crystal size) was added, and the mixture was placed at room temperature for 48 h. Finally, the NaCl was washed with deionized water to obtain the structure of BSFS. The BSFS was cut into a 2 cm × 2 cm × 3 mm cube shape. The trimmed BSFS was lyophilized and sterilized with ^60^Co radiation for further research. The silk film, silk sponge, and BSFS were cut into long strips (2 cm × 0.5 cm × 0.2 mm, 2 cm × 0.5 cm × 2.8 mm, 2 cm × 0.5 cm × 3 mm) and underwent mechanical testing using a universal mechanical testing machine (Instron, 68SC‐1, USA). Three samples were tested per group.

### 4.2 Preparation of the BAMMA

The bladder tissues were harvested from 5 to 6‐month‐old New Zealand white rabbits. The BAM was prepared using physical freeze–thaw and chemical detergents. The harvested bladder tissues were frozen at −80°C for at least 24 h and then thawed at room temperature. The bladder tissues were decellularized with 2% sodium dodecyl sulfate (SDS, Sigma) for 2 h, and then with deionized water for 1 h. Subsequently, 1% Triton X‐100 (Sigma) as a decellularized liquid was used for 4 h. Finally, the bladder tissues were rinsed with deionized water for 24 h to obtain the BAM. During the decellularization process, the decellularized solution was continuously stirred at a speed of 70 rpm. The BAM and native bladder were fixed in 10% formalin solution for 48 h, embedded in paraffin, and sectioned into 5 μm slices. Hematoxylin and eosin (H&E), Masson's trichrome (Masson), and 4,6‐diamidino‐2‐phenylindole (DAPI) staining were used to observe the decellularization effect. In addition, DNA quantification was used to assess the DNA residue. Native bladder tissues (*n* = 3) and BAM (*n* = 3) were dabbed dry with tissue paper and weighed. The DNA contents extracted using a Genomic DNA Kit (Tiangen, China) were measured using a Nanodrop ND3300 spectrophotometer (Thermo Fisher Scientific, USA). The DNA quantity was expressed as ng/mg wet weight of the samples. Furthermore, the prepared BAM was lyophilized and ground into powder. The BAM powder was solubilized through enzymatic digestion as previously described.^20^ Pepsin (Sigma) was dissolved in 0.1 M hydrochloric acid to make a concentration of 1 mg/mL. The 200 mg of BAM pieces were digested in 20 mL of pepsin solution under constant stirring for 48 h at 37°C to obtain a 10 mg/mL concentration of BAM solution. The pH of the BAM solution was adjusted to a range of 8 to 9 by adding 1 M NaOH. The methacrylic anhydride was added to the solution at 4°C, and the mixture was stirred continuously in the dark for 24 h. During the reaction, the pH was maintained between 8 and 9. Subsequently, the reaction solution was dialyzed against deionized water at 4°C for 2 days, followed by freezing and lyophilization to obtain the BAMMA. The prepared BAMMA was sterilized with ^60^Co radiation for further use.

### 4.3 Proton nuclear magnetic resonance ( ^1^H NMR) spectroscopy

The samples of BAM and BAMMA were placed into separate EP tubes, each containing ~1 mL of deuterium oxide, and then transferred to nuclear magnetic resonance (NMR) tubes. The ^1^H NMR test was performed using a 400/500 MHz Nuclear Magnetic Resonance spectrometer (Bruker, Avance NEO, Germany).

### 4.4 Rheological analysis of BAMMAH

The BAMMA was dissolved in PBS and lithium phenyl‐2,4,6‐trimethylbenzoylphosphinate (LAP, Sunp Biotech, China) to prepare BAMMA solutions with different concentrations of 0.5%, 1%, 2% and 4% (wt/vol). The different BAMMA solutions with LAP at a final concentration of 0.25% (vol/vol) were exposed to 405 nm UV light for 1 min to obtain the BAMMA hydrogels (BAMMAHs). The rheological properties of the BAMMAHs were tested using a Rheometer (Anton Paar MCR 302, Austria) with parallel‐plate geometry (20‐mm diameter, 0.2 mm gap). Evaluate the relationship between shear rate and viscosity of the four different BAMMAHs in the shear rate range of 1 to 1000 s^−1^ at the temperature of 37°C. Time sweep oscillatory tests were performed at 0.5% strain in the frequency range of 0.1 to 10 Hz. The storage modulus (G') and loss modulus (G”) of the four different BAMMAHs were measured.

### 4.5 ADSCs isolation, culture and identification

The ADSCs isolation and culture were prepared according to our previous study.^44^ The inguinal adipose tissues of 5 to 6‐month‐old New Zealand white rabbits were isolated and rinsed with PBS containing 100 U/mL penicillin and 100 μg/mL streptomycin. The adipose tissues were minced and digested with 1 mg/mL collagenase type IV (Sigma) and dispase II (Sigma) at 37°C for 30 min with gentle agitation. The enzymatic activity was then neutralized by adding an equal volume of Dulbecco's Modified Eagle Medium (DMEM, Gibco, USA) containing 10% fetal bovine serum (FBS, Gibco, USA). The dissociated tissue was filtered to remove debris and centrifuged at 1000 rpm for 10 min. The collected cells were resuspended and planted onto a 10‐cm culture dish in DMEM with 10% FBS, and then incubated at 37°C with 5% humidified CO_2_. The culture medium was changed every 3 days. Third passage ADSCs were used for flow cytometry identification. The ADSCs were digested with 0.25% trypsin‐0.02% EDTA, centrifuged at 1000 rpm for 5 min, and resuspended at a concentration of 10^6^ cells/ml in PBS. Cells were incubated in the dark for 45 min at 4°C with the following primary antibodies: CD29, CD44, CD34, and CD45. (antibodies information: Table S1). After incubation, the cells were washed three times with PBS and stored at 4°C prior to analysis using a FACSCalibur flow cytometer (Becton‐Dickinson, USA). Data was analyzed using CellQuest software.

### 4.6 Cytotoxicity test of the prepared BSFS‐BAMMAH

The BAMMA was dissolved in PBS and LAP to prepare 2% (wt/vol) BAMMA solution with the LAP at a final concentration of 0.25% (vol/vol). The 2% BAMMA solution (300 μL) was dropped onto the surface of the BSFS (2 cm × 2 cm × 3 mm), which was then exposed to 405 nm UV light for 1 min to obtain the BSFS‐BAMMAH. The prepared BSFS‐BAMMAH were immersed in DMEM at 37°C for 72 h, and the extract was collected for the cytotoxicity test. The third passage ADSCs were seeded in 96‐well plates at a concentration of 5000 cells/well, and cultured in DMEM (10% FBS) for 24 h at 37°C with 5% humidified CO_2_. The culture medium was replaced by the BSFS‐BAMMAH extract and the control DMEM, respectively. At 1, 3, and 5 days, the ADSCs were incubated with 10 μL of Cell Counting Kit‐8 (CCK‐8, Invitrogen) reagent in 5% humidified CO_2_ for 2 h at 37°C. The absorbance at 450 nm was measured by a microplate reader (Molecular Devices, Sunnyvale, CA). At the same time, live/dead staining was used to evaluate the viability of the ADSCs in the BSFS‐BAMMAH extract. At 1, 3, and 5 days, the ADSCs were washed twice with PBS and then incubated with 100 μL live/dead staining solution (Invitrogen) at 37°C for 15 min. After washing with PBS, the ADSCs were examined by Nikon Eclipse Ti2‐U fluorescence microscope (Nikon, Tokyo, Japan) to distinguish the live and dead cells.

### 4.7 Differentiation of ADSCs into VECs and NCs

Induction scheme of VECs. The third passage of ADSCs was plated onto 6‐well plates coated with 0.1% gelatin (Sigma) at a concentration of 5 × 10^4^ cells per well and cultured in DMEM (10% FBS) for 24 h at 37°C with 5% humidified CO_2_. Subsequently, endothelial induction medium (EIM) was used to culture ADSCs for 10 days to differentiate them into VECs, and the medium was changed every other day. EIM contains endothelial cell‐specific culture medium (Meisen, China), 50 ng/mL VEGF (Chamot Biotech, China), 10 ng/mL bFGF (PeproTech, USA), and 1% penicillin/streptomycin (Invitrogen). Induction scheme of NCs. The third passage ADSCs were plated onto 6‐well plates coated with poly‐L‐lysine (Thermo Fisher Scientific, USA) at a concentration of 5 × 10^4^ cells per well and cultured in DMEM (10% FBS) for 24 h at 37°C with 5% humidified CO_2_. Neural pre‐induction medium (NPIM) was used to culture ADSCs for 1 day and then switched to the neural induction medium (NIM) for 9 days to differentiate into NCs, and the medium was changed every other day. NPIM contains Neurobasal culture medium (Gibco), 2% B27 (Gibco), 1% FBS, 20 ng/mL EGF (PeproTech, USA), 20 ng/mL bFGF, and 1% penicillin/streptomycin. NIM contains Neurobasal culture medium (Gibco), 2% B27 (Gibco), and 1% penicillin/streptomycin.

### 4.8 Immunofluorescence staining

The cells were washed three times with PBS and then fixed in 4% paraformaldehyde for 30 min at room temperature, followed by washing three times in PBS. The cells were then permeabilized for 10 min in 0.3% Triton X‐100, and then blocked for 30 min with 5% goat serum at 37°C. The cells were incubated respectively with anti‐CD31 rabbit polyclonal antibody (1:500, Proteintech) and anti‐β‐III Tubulin mouse monoclonal antibody (1:500, Proteintech) overnight at 4°C. After several washes, the cells were then incubated with secondary antibodies against rabbit or mouse tagged with Cyanine 3 (Cy3; red) for 2 h at room temperature. The nuclei were counterstained with DAPI. The photomicrographs were observed with a Nikon Eclipse Ti2‐U fluorescence microscope.

### 4.9 Reverse transcription quantitative polymerase chain reaction (RT‐qPCR)

Total RNA was extracted using TRIzol Reagent (Sigma, USA). The total RNA concentration was determined using the Nanodrop ND3300 spectrometer (Thermo Fisher Scientific, USA). RT‐qPCR was performed using the PrimeScript RT reagent Kit (TaKaRa). The expression of genes was normalized to GAPDH. All reactions were repeated in triplicate. The primer sequences are shown in Table S2.

### 4.10 Constructing the engineered bladder patch through a layer‐by‐layer assembly technique

The 2% (wt/vol) BAMMA solution (100 μL) and 0.25% (vol/vol) LAP mixed with ADSCs (1 × 10^6^ cells) and NCs (1 × 10^5^ cells) were dropped on the surface of the BSFS, which was exposed to 405 nm UV light for 1 min to obtain the BAMMAH layer containing ADSCs and NCs as the bottom layer. Subsequently, the 2% (wt/vol) BAMMA solution (100 μL) and 0.25% (vol/vol) LAP mixed with ADSCs (1 × 10^6^ cells) and VECs (1 × 10^5^ cells) were dropped on the surface of the BSFS, which was exposed to 405 nm UV light for 1 min to obtain the BAMMAH layer containing ADSCs and VECs as the middle layer. Moreover, the 2% (wt/vol) BAMMA solution (100 μL) and 0.25% (vol/vol) LAP mixed with ADSCs (1 × 10^6^ cells) were dropped on the surface of the BSFS, which was exposed to 405 nm UV light for 1 min to obtain the BAMMAH layer only containing ADSCs as the upper layer. The tissue engineering bladder patch with a three‐layer structure (BSFS‐BAMMAH‐ADSCs‐VECs‐NCs) was constructed through the layer‐by‐layer assembly technique. In addition, only ADSCs were used as seed cells to construct the engineered bladder patch (BSFS‐BAMMAH‐ADSCs) in the control group. The 2% (wt/vol) BAMMA solution (300 μL) and 0.25% (vol/vol) LAP were mixed with ADSCs (3 × 10^6^ cells) and dropped onto the surface of the BSFS, which was then exposed to 405 nm UV light for 1 min to obtain the engineered bladder patch. The constructed engineered bladder patches were cultured in a 6‐well plate, and 5 mL of DMEM containing 10% FBS was slowly added to the plate. The culture medium was changed every 2 days. After 3 days of cultivation, the constructed engineered bladder patch (BSFS‐BAMMAH‐ADSCs‐VECs‐NCs) was fixed in 10% formalin solution for 48 h, embedded in paraffin, and sectioned into 5 μm slices. The slices were stained with H&E and DAPI to observe the distribution of the ADSCs in the patch.

### 4.11 Scanning electron microscopy

The constructed engineered bladder patch (BSFS‐BAMMAH‐ADSCs‐VECs‐NCs) was fixed with 2.5% glutaraldehyde for 2 h at room temperature, washed twice with PBS, and then dehydrated using a gradient of alcohols. It was subsequently lyophilized. The silk film, silk sponge, BSFS, BAMMAHs, and engineered bladder patch (BSFS‐BAMMAH‐ADSCs‐VECs‐NCs) were lyophilized and coated with 2 μm AuPd using a sputter coater system (sputter module 108auto, Cressington Scientific, Watford, UK), respectively. Micrographs were captured with a scanning electron microscope (Nova200 Nanolab).

### 4.12 Animal experiments

All animal experiments were approved by the Ethics Committee of the Chinese PLA Air Force Medical Center (No. 2024‐44‐PJ01). All the rabbits undergoing surgery were induced into anesthesia by intramuscular injection of Zoletil™50 (4 mg/kg), and Xylazine (8 mg/kg), and isoflurane (2%) was administered to maintain anesthesia during the surgery. Thirty‐three male New Zealand white rabbits (2–4 kg, 5–6 months old) were randomly divided into three groups: the engineered bladder patch with a three‐layer structure (BSFS‐BAMMAH‐ADSCs‐VECs‐NCs) (Experimental group, *n* = 12), the engineered bladder patch with a single‐layer structure (BSFS‐BAMMAH‐ADSCs) (Control group, *n* = 12), and the cystotomy group (Normal group, *n* = 9). After 3 days of cultivation, the engineered bladder patches from the experimental and control groups were sutured onto the omentum, respectively. Among them, the sponge surface of the experimental group and control group was faced toward the omentum, and the film surface toward the peritoneal cavity. After 1 week, three rabbits from each group were sacrificed, and the incubated scaffolds were harvested and fixed in 10% neutral buffered formalin for histological examination. The other engineered bladder patches incubated with omentum were used for bladder augmentation.

### 4.13 Bladder augmentation

The rabbits were anesthetized, and the surgical area was disinfected with iodophor. The sterile sheet was spread. A 2 cm incision along the low abdominal midline was made, and the underlying tissue was sequentially dissected to expose the bladder tissue. A 2 cm × 2 cm full‐thickness square defect was made at the dome of the bladder. The engineered bladder patches of the experimental group and control group, incubated with omentum, were used to repair this defect, respectively. The bladder defect was marked with non‐absorbable 5–0 silk braided sutures at the four corners and then anastomosed by absorbable 5–0 sutures. In the normal group, the excised full‐thickness square bladder tissues were used to repair the bladder defect. The watertight seal was confirmed by filling the repaired bladder with sterile saline via the double‐lumen Foley Catheter (6Fr). Finally, the incision was sutured layer by layer. The rabbits were sacrificed at 2, 4, 12 weeks after bladder augmentation (*n* = 3 for each time point).

### 4.14 Retrograde cystography and cystoscopy

The cystography and cystoscopy were performed under anesthesia. At each time point, the bladder was emptied, and the contrast medium (iohexol) was injected into the bladder through a double‐lumen Foley Catheter (6Fr). X‐ray film was obtained from each bladder. Ureterorenoscope (6.3/3.6Fr, Shenzhen HugeMed Medical) was used for cystoscopy in rabbits.

### 4.15 Component analysis of bladder stones

The removed bladder stones were washed with distilled water and dried in a 60°C oven. The stones mixed with the potassium bromide at a ratio of 1:100 were pressed into a transparent sheet with a thickness of about 1 mm in a tablet press. The transparent sheet was placed in the spectrometer (Lambda, LIIR‐20, China), and the sample spectrum was measured at room temperature. The stone's composition was obtained by comparing it with the standard spectrum library.

### 4.16 Urodynamic studies

Bladder urodynamic studies were measured at 12 weeks post‐implantation, as described in the previous protocol.^4^ Briefly, the bladder was emptied through a double‐lumen Foley Catheter (6Fr). The infusion pump connected to the tapered connector filled the bladder with warm sterile saline at a rate of 7.5 mL/min. The intravesical pressure was recorded simultaneously by a pressure‐monitoring device that was connected to the other end of the double‐lumen Foley Catheter with the valve removed and balloon punctured. The urodynamic curves and parameters, including bladder capacity, bladder peak pressure, and the bladder compliance (dividing the instilled volume by bladder peak pressure), were recorded and analyzed.

### 4.17 Histological and immunofluorescence analysis

At 2, 4 and 12 weeks after implantation, the rabbits were euthanized, and the bladders were excised to prepare paraffin sections. Sections (4 μm) were stained with H&E. For immunofluorescence analysis, sections were deparaffinized, blocked, and incubated with primary antibodies against cytokeratin (CK) AE1/AE3, α‐smooth muscle actin (α‐SMA), CD31, and β‐III Tubulin at 4°C overnight (antibodies information: Table S1). Afterwards, the sections were incubated with species‐matched secondary antibodies (FITC or Cy3), and the nuclei were counterstained with DAPI. Photomicrographs were acquired by a Nikon Eclipse 80i fluorescence microscope. Photometric analyses, including positive area of AE1/AE3, α‐SMA, β‐III Tubulin, and the CD31‐positive blood vessel numbers, were performed based on 6 randomly selected fields from 3 slides by ImageJ 1.51 software (National Institutes of Health).

### 4.18 Statistical analysis

Statistical analysis was performed using SPSS (27.0, USA). The results were expressed as mean ± standard deviation. The differences between groups were estimated using a two‐tailed Student's *t*‐test or a one‐way analysis of variance with Bonferroni post hoc test. *p*‐values of less than 0.05 were considered statistically significant.

## 5 CONCLUSION

The prepared BSFS combined the rigidity of SF film and the elasticity of SF sponge, providing a support framework for the construction of engineered bladder and confirming the stability of the BSFS preparation system. Moreover, the prepared photo‐BAM hydrogel had a rapid gelling property and could be used as a carrier for seed cells to rapidly construct a multi‐layer engineered bladder. Furthermore, the neural and vascular endothelial induction schemes explored by our team were feasible and reliable. In addition, the engineered bladder patch constructed by layer‐by‐layer assembly technology regenerated blood vessels and neural networks internally, achieving the regeneration of bladder wall structure and the restoration of bladder physiological function, providing a feasible method for constructing the engineered bladder with a blood vessel network and neural innervation.

## AUTHOR CONTRIBUTIONS

**Pengchao Wang:** Methodology; writing – original draft. **Kaipeng Bi:** Validation; visualization; writing – original draft. **Zheng Wang:** Software; visualization. **Jinxuan Zhang:** Data curation; formal analysis. **Yang He:** Investigation; software. **Jianye Li:** Investigation; validation. **Linqing Ji:** Data curation; supervision. **Di Li:** Data curation; formal analysis; methodology. **Dawei Mu:** Supervision; writing – review and editing. **Ziyan An:** Investigation; methodology. **Shuwei Xiao:** Conceptualization; funding acquisition; resources; writing – review and editing. **Weijun Fu:** Conceptualization; funding acquisition; supervision. **Fei Liu:** Project administration; supervision.

## FUNDING INFORMATION

This study was supported by Beijing Natural Science Foundation (7244413); The Youth Project of the Talent Cultivation Plan of the Chinese PLA Air Force Medical Center (2025RCQN10); National Natural Science Foundation of China (82270721), and Beijing Natural Science Foundation (7252169).

## CONFLICT OF INTEREST STATEMENT

The authors declare no conflict of interest.

## Supporting information


**Figure S1.** Evaluation of the prepared BAM. (A) H&E, Masson's trichrome, and DAPI staining of the native bladder tissue and BAM; H&E and Masson staining, scale bar = 100 μm; DAPI staining, scale bar = 200 μm. (B) The DNA contents of the native bladder tissue and BAM (*n* = 3). (*p*‐values were calculated using a two‐tailed Student's *t*‐test, *p < 0.05).
**Figure S2.** Flow cytometry identification of the isolated ADSCs.
**Figure S3.** Evaluation of the constructed engineered bladder patch in the experimental group. (A) H&E staining, scale bar = 200 μm. (B) DAPI staining, scale bar = 200 μm. (C) SEM micrograph, scale bar = 5 μm.
**Table S1.** Detailed antibodies information.
**Table S2.** Primer sequence.

## Data Availability

The data that support the findings of this study are available from the corresponding author upon reasonable request.
