# Supplementary material for: The engineered bladder patch with a three‐layer structure promotes the regeneration and functional recovery of the bladder in a rabbit model
Source: Bioeng Transl Med. 2026 Apr 21;11(4):e70140. doi: 10.1002/btm2.70140 (PMC13327650; doi:10.1002/btm2.70140)
Supplement: Supplementary file 1 — Figure S1. Evaluation of the prepared BAM. (A) H&E, Masson's trichrome, and DAPI staining of the native bladder tissue and BAM; H&E and Masson staining, scale bar = 100 μm; DAPI staining, scale bar = 200 μm. (B) The DNA contents of the native bladder tissue and BAM (n = 3). (p‐values were calculated using a two‐tailed Student's t‐test, *p < 0.05). Figure S2. Flow cytometry identification of the isolated ADSCs. Figure S3. Evaluation of the constructed engineered bladder patch in the experimental group. (A) H&E staining, scale bar = 200 μm. (B) DAPI staining, scale bar = 200 μm. (C) SEM micrograph, scale bar = 5 μm. Table S1. Detailed antibodies information. Table S2. Primer sequence. [file BTM2-11-e70140-s001.docx]

**
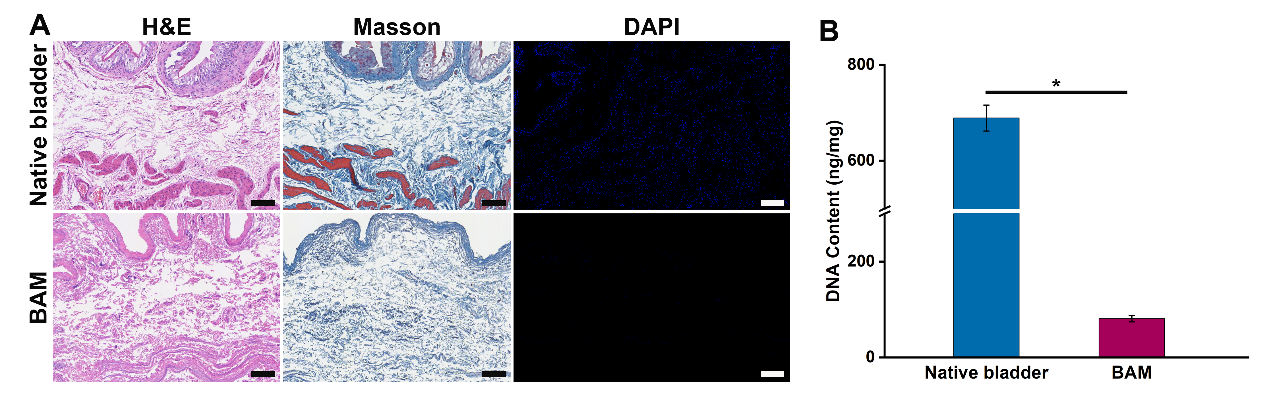
**

**Figure S1.** Evaluation of the prepared BAM. (A) H&E, Masson’s trichrome, and DAPI staining of the native bladder tissue and BAM; H&E and Masson staining, scale bar = 100 μm; DAPI staining, scale bar = 200 μm. (B) The DNA contents of the native bladder tissue and BAM (n = 3). (*P*-values were calculated using a two-tailed Student's t test, ^*^*P* < 0.05)


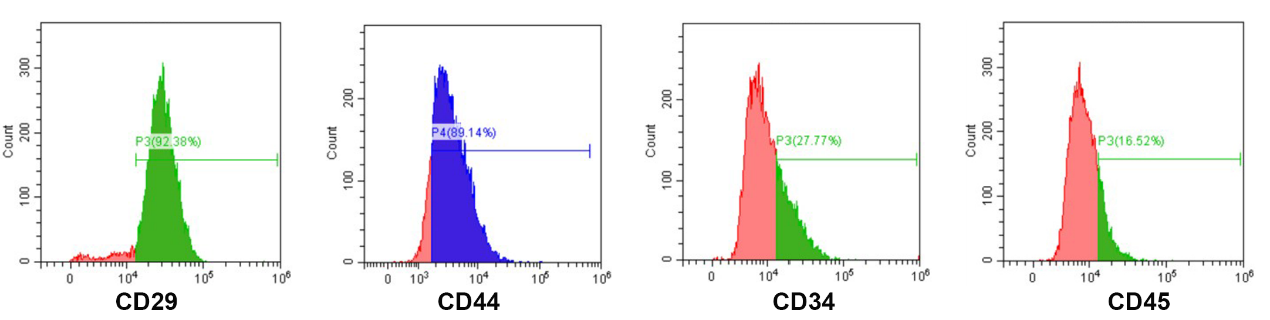


**Figure S2.** Flow cytometry identification of the isolated ADSCs.


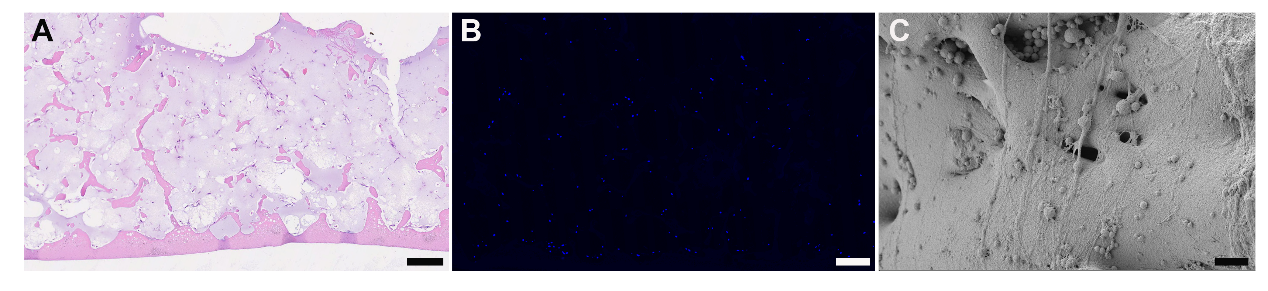


**Figure S3.** Evaluation of the constructed engineered bladder patch in the experimental group. (A) H&E staining, scale bar = 200 μm. (B) DAPI staining, scale bar = 200 μm. (C) SEM micrograph, scale bar = 5 μm.

**Table S1** **Detailed antibodies information**

| Against | catalog | Dilution |
| --- | --- | --- |
| CD29-FITC | bs-0486R, Bioss Inc | 1:100 |
| CD44-FITC | bs-0521R, Bioss Inc | 1:100 |
| CD34-FITC | GTX75411, GeneTex | 1:10 |
| CD45-PE | GTX01462-08, GeneTex | 1:20 |
| AE1/AE3 | AM10028PU-S, OriGene Technologies, USA | 1:100 |
| α-SMA | 14395-1-AP, Proteintech, China | 1:200 |
| CD31 | 11265-1-AP, Proteintech, China | 1:200 |
| β-Ⅲ Tubulin | 66375-1-lg, Proteintech, China | 1:200 |

**Table S2** **Primer sequence**

| mRNA | Primer sequence |
| --- | --- |
| GAPDH | Forward primer: ACAGGCAGCCGCTTCTTCTC |
|  | Reverse primer: TCCGTTCACTCCGACCTTCAC |
| CD31 | Forward primer: GGTTCTGAAGGTCAAGGTGATAGC |
|  | Reverse primer: GAGCAGCGGAGCACAATGTC |
| KDR | Forward primer: TACACCGTCATCCTCACCAACC |
|  | Reverse primer: GAGTCCACAGGCGAGATCAGAG |
| Nestin | Forward primer: CTGCGTGCCACTGAGAACTTC |
|  | Reverse primer: AGCTGCTGCCGACCTTCC |
| β-Ⅲ Tubulin | Forward primer: CTGAGAGCAACATGAACGACCTG |
|  | Reverse primer: CCTCGGACTCTTCTTCATCATCTTC |
